# Real-world experience with monthly and quarterly dosing of fremanezumab for the treatment of patients with migraine in Japan

**DOI:** 10.3389/fneur.2023.1220285

**Published:** 2023-07-06

**Authors:** Shiho Suzuki, Keisuke Suzuki, Tomohiko Shiina, Yasuo Haruyama, Koichi Hirata

**Affiliations:** ^1^Department of Neurology, Dokkyo Medical University, Mibu, Tochigi, Japan; ^2^Integrated Research Faculty for Advanced Medical Sciences, Dokkyo Medical University, Mibu, Tochigi, Japan

**Keywords:** migraine, migraine days per month, fremanezumab, calcitonin gene-related peptide, real-world study

## Abstract

**Background:**

The effectiveness of fremanezumab in treating migraine has been demonstrated in randomized controlled trials. However, real-world study results are still limited.

**Methods:**

We conducted a single-center, observational study that included patients with episodic migraine (EM) and chronic migraine (CM) who received fremanezumab monthly or quarterly over 6-month periods. The primary outcome of this study was to evaluate changes in monthly migraine days (MMD) and responder achievement after treatment with fremanezumab. The secondary aim was to characterize the predictors of responder at 6 months. We also evaluated the effectiveness of fremanezumab in the patients who switched from other calcitonin gene-related peptide (CGRP) monoclonal antibodies, and compared the effectiveness of fremanezumab between the monthly and quarterly dosing groups. One hundred twenty-seven patients with migraine (age, 45.2 ± 12.6 years; 96 women) who received at least one dose of fremanezumab with ≥3 months of follow-up were included. The number of MMD was assessed by headache diary.

**Results:**

The changes in MMD from baseline at 1, 3, and 6 months were −6.1 ± 4.7, −7.7 ± 4.4, and − 8.5 ± 4.5 days in the total cohort, respectively (*p* < 0.001). The ≥50%, ≥ 75 and 100% responder rates at 6 months were 67.6, 22.5, and 5.4% in the total cohort, 90.4, 36.5, and 9.6% in the EM group, and 52.2, 14.9, and 1.5% in the CM group, respectively. Fremanezumab was also effective in 35 patients who switched from other CGRP monoclonal antibodies. Quarterly and monthly fremanezumab doses were equally effective in MMD reduction in the EM and CM groups. In the CM group, 65.1% experienced remission to EM after 6 months. Adverse reactions were mild and occurred in 9.5% of total patients. An at least ≥50% reduction in MMD from months 1 to 3 better predicted a ≥ 50% reduction in MMD at 6 months with 90.5% sensitivity and 80.6% specificity (*p* < 0.001).

**Conclusion:**

In our real-world study, quarterly and monthly fremanezumab dosing showed both favorable effectiveness and tolerability in patients with migraine.

## Introduction

Migraine is a common and highly disabling neurological disorder that affects more than 1 billion people worldwide, with a prevalence of 15% per year and peaking among those aged 35–39 years (1). Migraine negatively affects many aspects of patients’ daily lives, including careers, parenting, and partnerships (1). In a recent online survey using medical claims data, which included data on 21,480 individuals, 53% of individuals with migraine had severe pain, and 73% of those reported “moderate or severe” impairment in activities of daily living (2). In addition, migraine patients frequently experience symptoms other than headache, including premonitory symptoms, aura, sensory hypersensitivities, and accompanying symptoms, during headache attacks and interictal periods, which interfere with the patients’ daily lives. Based on these observations, appropriate diagnosis and treatment of migraine is an imperative issue. Patients with episodic migraine with low attack frequency or low disabling intensity can be managed with acute medication alone. However, preventive headache treatment is recommended when, despite acute treatment, the patient’s daily life is considerably disturbed or when migraine occurs more than 4 days per month (3).

Calcitonin gene-related peptide (CGRP) plays an important role in the pathophysiology of migraine, and the use of several CGRP monoclonal antibodies, recently available as migraine-specific prophylactic agents, has begun to substantially improve the quality of life of patients with migraine (4–6). Fremanezumab is a humanized monoclonal antibody that selectively targets the alpha and beta isoforms of CGRP, and it is approved for prophylaxis of migraine in adults. This drug is administered in doses of 225 mg monthly or 675 mg quarterly, depending on the patient’s preference and the physician’s recommendation. Additionally, the efficacy of fremanezumab has been demonstrated in several real-world studies in Europe and the United States (7–9). Barbanti et al. reported the effectiveness of fremanezumab in patients with chronic and high-frequency episodic migraine in multicenter real-world studies for 3 and 6 months (7, 10). However, the clinical evidence for fremanezumab in migraine prophylaxis in Asia is limited to randomized controlled trial (RCT) results and *post hoc* analyses (11–14), and more clinical evidence from real-world studies is needed, especially in Asian migraine patients with various clinical backgrounds.

This study provides 6-month real-world evidence from a single center on the efficacy and safety of monthly or quarterly fremanezumab in treating episodic migraine (EM) and chronic migraine (CM). The primary outcome of this study was to evaluate changes in monthly migraine days (MMD) and responder achievement after treatment with fremanezumab. The secondary aim was to characterize the predictors of responder at 6 months. We also evaluated the effectiveness of fremanezumab in the patients who switched from other CGRP monoclonal antibodies, and made the comparison of the effectiveness of fremanezumab between the monthly and quarterly dosing groups.

## Methods

### Study design

We conducted a 6-month retrospective, observational, single-center cohort study among patients with episodic or chronic migraine who attended our headache outpatient clinic and received monthly or quarterly doses of fremanezumab.

### Patients

**Figure 1:**
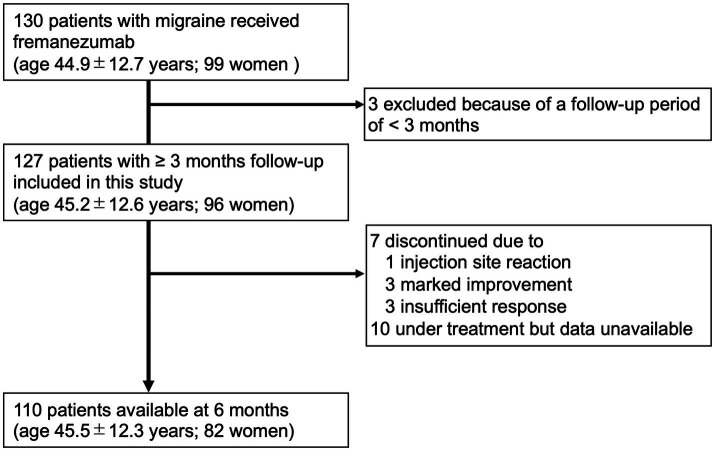
Study flowchart.

Among the patients attending our outpatient headache clinic from April 2022 to January 2023, 130 adult patients with EM or CM (age, 44.9 ± 12.7 years; 99 women) received at least one dose of fremanezumab. All patients in this study received fremanezumab injections using prefilled syringes. Patients had at least 2 months of treatment with one or more prophylactic medications, including other types of CGRP monoclonal antibodies, prior to starting either monthly (225 mg) or quarterly (675 mg) doses of fremanezumab. The inclusion criteria for this study were adults with a confirmed diagnosis of migraine and at least 3 months of follow-up available after treatment with fremanezumab. The exclusion criteria were failure to keep a headache diary, age less than 18 years, and organic brain lesions that could affect headache. Finally, 127 patients with migraine (age 45.2 ± 12.6 years; 96 women; 73 with CM) were included in this study (Figure 1). Considering the nature of the real-life clinical study, complications such as medication overuse headache (MOH) and psychiatric disorders were also included. The patients included CGRP-naive subjects and those switching from other CGRP monoclonal antibodies to fremanezumab.

### Diagnosis of migraine

Migraine with and without aura was diagnosed by headache specialists according to the latest International Classification of Headache Disorders 3rd edition (ICHD-3) (15). CM was defined as headache that lasted at least 15 days per month for at least 3 months during which migraine features were in evidence at least 8 days per month, and EM was defined as headache that occurred for 4–14 days per month. MOH was diagnosed according to ICHD-3 (15).

### Clinical assessments

The number of MMD before and 1–6 months after fremanezumab treatment was obtained from headache diaries. Based on the headache diary, a reduction of ≥50%, ≥75%, or 100% in MMD from baseline was calculated and was defined as ≥50%, ≥75%, or 100% response at 1, 3 and 6 months, respectively. Data regarding the duration of migraine, comorbidities, previous prophylactic medications, presence or absence of aura and accompanying symptoms, body mass index, and migraine characteristics were obtained from clinical records. Among patients with CM, the rate of remission to EM was assessed. Adverse reactions were also investigated from clinical records regarding safety after fremanezumab administration.

### Ethics approval

This study was performed in accordance with the Declaration of Helsinki guidelines and approved by the Institutional Review Board of Dokkyo Medical University. All participating patients were informed about this observational study in an outpatient setting and had the opportunity to opt out of participation in the study. Our Institutional Review Board waived the need for patients to sign informed consent forms based on the retrospective, observational nature of the study.

### Statistical analysis

Sample size calculations were not performed given the real-world setting of this study. The Mann–Whitney *U* test or Student’s *t* test was used for comparison of continuous variables as appropriate, and the chi-square test or Fisher’s exact test (when the expected frequency of less than 5 exceeded 20%) was used for comparison of categorical variables. A generalized linear mixed-effects model (GLMM) followed by Bonferroni’s *post hoc* test was performed to determine if there was a significant difference from baseline to each month in MMD after treatment with fremanezumab in the total cohort and in the EM and CM groups. The effect of monthly and quarterly dosing on MMD reduction in the EM and CM groups was analyzed by GLMM with repeated measures followed by a global test. Sensitivity and specificity of the ≥50% response in month 6 was calculated using each ≥50% response or combination of such responses at months 1–3. We also used a logistic regression model to analyze the association between a ≥ 50% response in month 6 and each ≥50% response or combination of such responses at months 1 to 3 after adjustment for related confounding factors.

Two-tailed *p* < 0.05 was considered statistically significant. IBM SPSS Statistics version 28 (IBM SPSS, Tokyo, Japan) was used for all statistical analyses. GraphPad Prism for Mac (Version 8; GraphPad Software, San Diego, United States) and Microsoft Excel version 16.18 were used to create figures.

## Results

### Participants

Data were available for 127 patients from baseline to 3 months, 112 patients at 4 months, 110 patients at 5 months, and 110 patients at 6 months.

### Clinical characteristics

Table 1 shows the baseline characteristics of patients with migraine. The total cohort consisted of 54 EM (30 monthly and 24 quarterly fremanezumab) and 73 CM (45 monthly and 29 quarterly fremanezumab). There were no significant differences in age, sex, aura status, or the presence of hypersensitivity symptoms, such as photophobia, phonophobia, osmophobia, nausea or allodynia, between the EM and CM groups. However, the duration of migraine was longer and the percentage of patients with MOH and the number of prophylaxes used in the past were higher in the CM group than in the EM group. Pain location did not differ between the EM and CM groups, but a pulsating nature was more common in the EM group. Baseline MMD was significantly greater in the CM group than in the EM group (21.7 ± 4.8 vs. 10.6 ± 2.5 days). In total, 35 (27.6%) patients switched to fremanezumab from other CGRP monoclonal antibodies (15 EM and 20 CM). Comorbidities were present in 54.3% of the total cohort, and cardiovascular disorder was more common in the CM group than in the EM group (28.8% vs. 13.0%; Supplementary Table 1). The percentages of patients with any comorbidity or two or more comorbidities were higher in the CM group than in the EM group (Table 1).

**Table 1:** Baseline characteristics of patients with migraine.

	Total	EM	CM	*p* value
n (M/F)	127 (31/96)	54 (14/40)	73 (17/56)	0.732
Fremanezumab dozing, monthly/quarterly	75/52	30/24	45/28	0.490
Age, years	45.2 ± 12.6	44.1 ± 12.7	46.0 ± 12.6	0.391
Body mass index (kg/m^2^)	22.5 ± 3.8	22.2 ± 3.9	22.8 ± 3.7	0.389
Migraine with aura, n (%)	26 (20.5)	15 (27.8)	11 (15.1)	0.079
Medication overuse headache, n (%)	18 (14.2)	0 (0.0)	18 (24.7)	**<0.001**
Disease duration, years	25.9 ± 11.5	23.3 ± 10.8	27.8 ± 11.7	**0.028**
Pain location, n (%)				
Unilateral	84 (66.1)	33 (61.1)	51 (69.9)	0.303
Bilateral	92 (72.4)	40 (74.1)	52 (71.2)	0.723
Pain characteristics, n (%)				
Pulsating	114 (89.8)	52 (96.3)	62 (84.9)	**0.037**
Pressing	82 (64.6)	34 (63.0)	48 (65.8)	0.745
Others	4 (3.1)	1 (1.9)	3 (4.1)	0.471
Sensory hypersensitivity, n (%)				
Photophobia	103 (81.1)	46 (85.2)	57 (78.1)	0.312
Phonophobia	94 (74.0)	40 (74.1)	54 (74.0)	0.990
Osmophobia	61 (48.0)	31 (57.4)	30 (41.1)	0.069
Nausea	115 (90.6)	52 (96.3)	63 (86.3)	0.057
Allodynia	25 (19.7)	12 (22.2)	13 (17.8)	0.536
Number of preventive medication classes used previously, n (%)				**0.012**
1	37 (29.1)	20 (37.0)	17 (23.3)	
2	44 (34.6)	23 (42.6)	21 (28.8)	
3	27 (21.3)	8 (14.8)	19 (26.0)	
4	10 (7.9)	3 (5.6)	7 (9.6)	
≥5	9 (7.1)	0 (0.0)	9 (12.3)	
Baseline MMD, n (%)	17.0 ± 6.8	10.6 ± 2.5	21.7 ± 4.8	**<0.001**
Comorbidities, n (%)	69 (54.3)	20 (37.0)	49 (67.1)	**<0.001**
≥2, n (%)	60 (47.2)	19 (35.2)	41 (56.2)	**0.019**

EM, episodic migraine; CM, chronic migraine; MMD, monthly migraine days.

Significant differences in *p* value are indicated in bold.

### Efficacy

#### Total cohort

**Figure 2:**
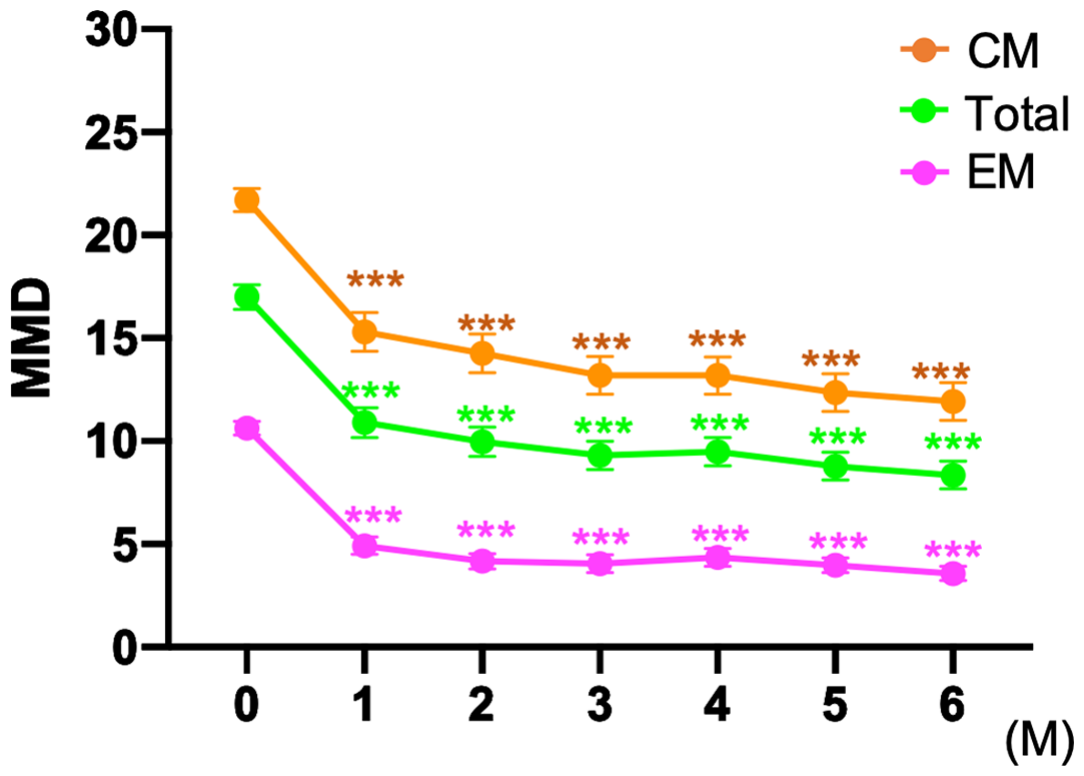
Mean number of monthly migraine days at baseline and after fremanezumab treatment ***p < 0.001, compared to baseline using a generalized mixed-effects model with repeated measures followed by Bonferroni’s multiple comparison test. MMD, monthly migraine days; EM, episodic migraine; CM, chronic migraine.

**Figure 3:**
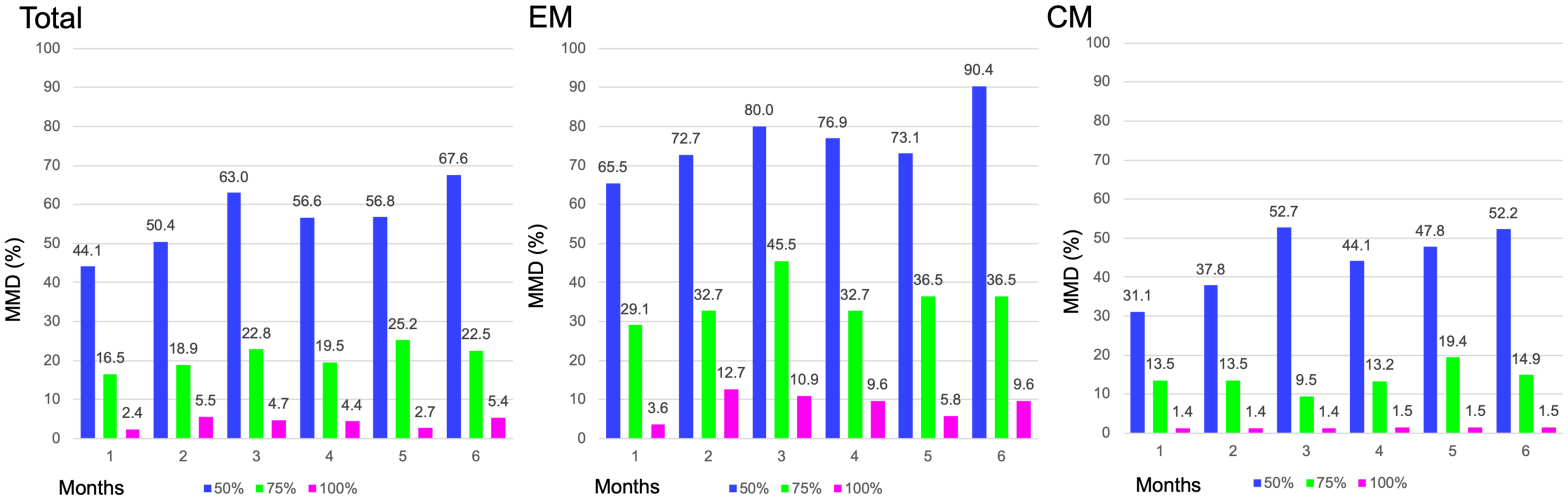
Proportion of patients achieving ≥ 50%, ≥75 and 100% reduction in MMD. MMD, monthly migraine days; EM, episodic migraine; CM, chronic migraine.

In the entire cohort, the baseline MMD value was 17.0 ± 6.8 days. After fremanezumab treatment, MMD changed by −6.1 ± 4.7, −7.7 ± 4.4, and − 8.5 ± 4.5 days at 1, 3, and 6 months, respectively (*p* < 0.001). Compared to baseline, the MMDs decreased significantly after 1–6 months of treatment with fremanezumab according to analysis using the GLMM with repeated measures followed by Bonferroni multiple comparison test (Figure 2). Overall, the ≥50% response rates at 1, 3, and 6 months were 44.1, 63.0, and 67.6%; the ≥75% response rates were 16.5, 22.8, and 22.5%; and the 100% response rates were 2.4, 4.7, and 5.4%, respectively (Figure 3).

#### EM group

The patients in the EM group had a baseline MMD value of 10.6 ± 2.5 days. After initiation of fremanezumab, the MMD reduction at 1, 3, and 6 months was −5.7 ± 2.7, −6.5 ± 2.7, and − 6.9 ± 2.3 days, respectively (*p* < 0.001). There was a significant reduction in MMD values after 1 to 6 months of treatment with fremanezumab compared to baseline according to analysis using GLMM with repeated measures followed by the Bonferroni multiple comparison test (Figure 2). In the EM group, the ≥50% response rates at 1, 3, and 6 months were 65.5, 80.0, and 90.4%; the ≥75% response rates were 29.1, 45.5, and 36.5%; and the 100% response rates were 3.6, 10.9, and 9.6%, respectively (Figure 3).

#### CM group

The baseline MMD value in the CM group was 21.7 ± 4.8 days. In the CM group, MMD decreased by −6.4 ± 5.7, −8.5 ± 5.1, and − 9.7 ± 5.3 days at 1, 3, and 6 months, respectively (*p* < 0.001). Significant decreases in the MMD value occurred after 1 to 6 months of fremanezumab treatment compared to baseline according to analysis using the GLMM with repeated measures followed by Bonferroni multiple comparison test (Figure 2). In the CM group, the ≥50% response rates at 1, 3, and 6 months were 31.1, 52.7, and 52.2%; the ≥75% response rates were 13.5, 9.5, and 14.9%; and the 100% response rates were 1.4, 1.4, and 1.5%, respectively (Figure 3).

#### Remission from CM to EM

Following fremanezumab treatment, 35 of 73 CM patients (48.0%) experienced remission to EM after 1 month. Of the remaining 38 CM patients, 13 (34.2%) remitted to EM after 3 months. In total, 65.1% of 63 CM patients remitted to EM after 6 months. Thirty-six (57.1%) of 63 CM patients had persistent EM remission between 4 and 6 months.

#### Clinical factors related to ≥50% response to fremanezumab at month 6

Patients were classified as ≥50% responders (≥50% 6 M) and < 50% responders at 6 months (<50% 6 M), and clinical characteristics were compared (Table 2). In the ≥50% 6 M group, there were lower baseline MMD values and a higher percentage of EM and nausea compared to the <50% 6 M group. The association between a ≥ 50% response rate from 1 to 3 months and a ≥ 50% response rate at 6 months was evaluated. Using at least one ≥50% response rate achieved in any of months 1–3 was a better predictor of a ≥ 50% response rate at 6 months (sensitivity, 90.5%; specificity, 80.6%; *p* < 0.001) than using ≥50% response rates from months 1, 2, and 3 alone or the average ≥ 50% response rate in months 1–3. A ≥ 50% response at 6 months was associated significantly with ≥50% response rates in months 1, 2, and 3 alone or any or the average ≥ 50% response rates in months 1, 2, and 3 in a multiple logistic model after adjustment for sex, age, EM or CM, and fremanezumab dosing (monthly or quarterly) (Table 3).

**Table 2:** Clinical factors related to a ≥ 50% reduction in MMD at 6  months.

	<50% 6 M	≥50% 6 M	*p* value
n (M/F)	36 (8/28)	74 (19/55)	0.693
Migraine diagnosis, EM/CM	5/31	42/32	**<0.001**
Fremanezumab dozing, monthly/quarterly	23/13	42/32	0.475
Age, years	45.2 ± 10.9	45.6 ± 12.9	0.882
Body mass index (kg/m^2^)	22.5 ± 3.8	22.5 ± 3.7	0.973
Migraine with aura, n (%)	6 (16.7)	18 (24.3)	0.362
Medication overuse headache, n (%)	9 (25.0)	8 (10.8)	0.053
Disease duration, years	25.9 ± 10.1	25.9 ± 5.8	0.987
Pain location, n (%)			
Unilateral	25 (69.4)	45 (60.8)	0.377
Bilateral	27 (75.0)	52 (70.3)	0.605
Pain characteristics, n (%)			
Pulsating	31 (86.1)	68 (91.9)	0.343
Pressing	25 (69.4)	46 (62.2)	0.454
Others	1 (2.8)	2 (2.7)	0.982
Sensory hypersensitivity, n (%)			
Photophobia	26 (72.2)	62 (83.8)	0.155
Phonophobia	28 (77.8)	57 (77.0)	0.930
Osmophobia	17 (47.2)	37 (50.0)	0.785
Nausea	29 (80.6)	69 (93.2)	**0.045**
Allodynia	7 (19.4)	12 (16.2)	0.674
Number of preventive medication classes used previously, n (%)			0.586
1	7 (19.4)	22 (29.7)	
2	13 (36.1)	24 (32.4)	
3	8 (22.2)	19 (25.7)	
4	4 (11.1)	5 (6.8)	
≥5	4 (11.1)	4 (5.4)	
Baseline MMD, n (%)	22.0 ± 5.8	14.4 ± 5.7	**<0.001**
Comorbidities, n (%)	18 (50.0)	40 (54.1)	0.689
≥2, n (%)	17 (47.2)	33 (44.6)	0.795

≥50% 6 M, ≥50% reduction in MMD at 6 months; <50% 6 M, <50% reduction in MMD at 6 months; MMD, monthly migraine days.

Significant differences in *p* value are indicated in bold.

**Table 3:** The prediction of ≥50% response rate at month 6 using ≥50% response rate from 1 to 3 months.

				Prediction of ≥ 50% 6 M					
	<50% 6 M	≥50% 6 M	*p* value	Sensitivity (%)	Specificity (%)	cOR	95% CI	aOR	95% CI
≥50% 1 M	3 (8.3)	45 (60.8)	**<0.001**	60.8	91.7	17.069	4.790–60.824	12.326	3.268–46.488
≥50% 2 M	3 (8.3)	51 (68.9)	**<0.001**	68.9	91.7	24.391	6.779–87.755	20.239	5.131–79.831
≥50% 3 M	6 (16.7)	63 (85.1)	**<0.001**	85.1	83.3	28.636	9.671–84.797	27.472	8.062–93.618
≥50% mean 1–3 M	4 (11.1)	50 (67.6)	**<0.001**	67.6	88.9	16.667	5.289–52.518	12.324	3.590–42.301
>50% any 1–3 M	7 (19.4)	67 (90.5)	**<0.001**	90.5	80.6	39.653	12.750–123.320	31.200	9.136–106.547

≥50% 1 M, ≥50% reduction in MMD at month 1; ≥50% 2 M, ≥50% reduction in MMD at month 2; ≥50% 3 M, ≥50% reduction in MMD at month 3; ≥50% 6 M, ≥50% reduction in MMD at month 6; ≥50% mean 1–3 M, average ≥ 50% reduction in MMD from 1 to 3 months; ≥50% any 1–3 M, at least one ≥ 50% reduction in MMD from 1 to 3 months; MMD, monthly migraine days; cOR, crude odds ratio; aOR, odds ratio adjusted for sex, age, episodic migraine or chronic migraine, fremanezumab dosing (monthly or quarterly); 95% CI, 95% confidence interval.

Significant differences in *p* value are indicated in bold.

#### Monthly versus quarterly dosing

**Figure 4:**
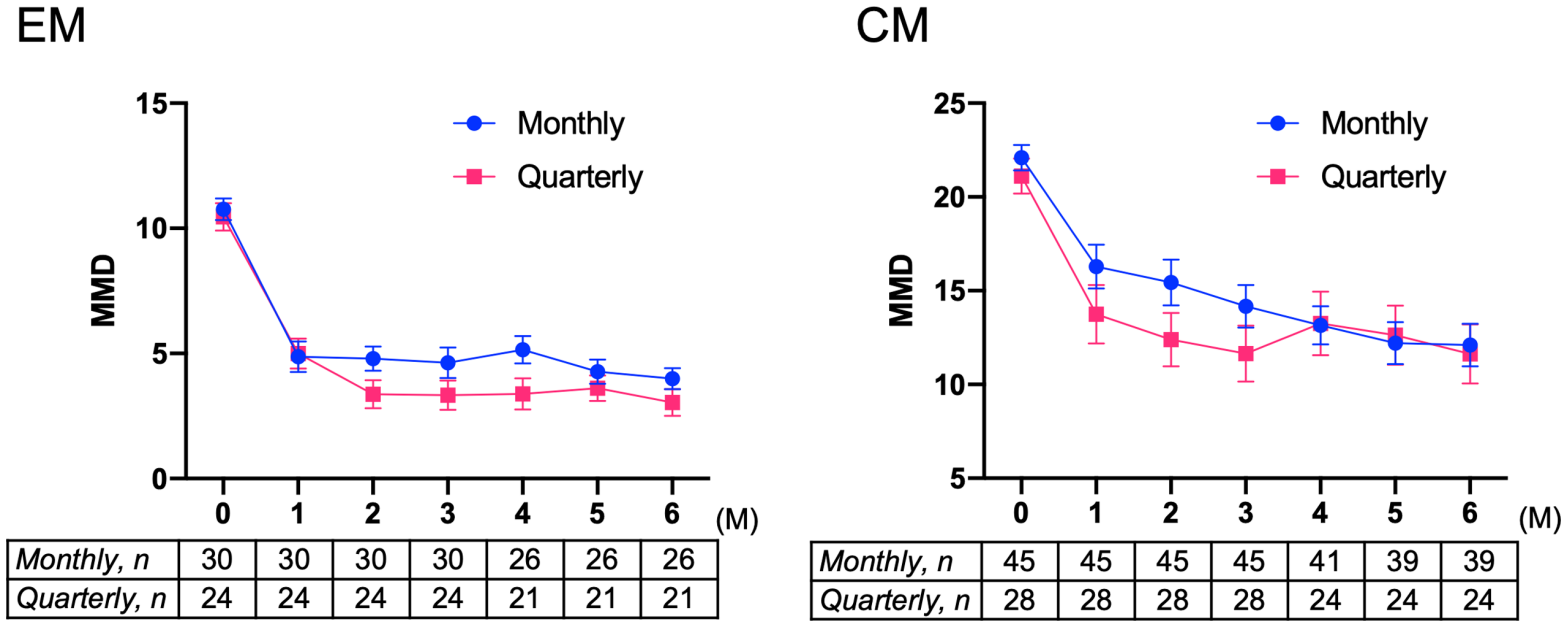
Mean changes from baseline in monthly migraine days in patients with EM or CM receiving monthly or quarterly doses of fremanezumab EM group: *F* = 104.2, *p* < 0.001 in time; *F* = 1.08, *p* = 0.304 in dosing; and *F* = 1.73, *p* = 0.223 in interaction between time and dosing. CM group: *F* = 82.98, *p* < 0.001 in time; *F* = 1.11, *p* = 0.295 in dosing; and *F* = 1.72, *p* = 0.116 in interaction between time and dosing. A generalized mixed-effects model with repeated measures followed by a global test was used. MMD, monthly migraine days; EM, episodic migraine; CM, chronic migraine.

Baseline characteristics of patients with migraine receiving fremanezumab monthly and quarterly are shown in Supplementary Table 2. There were no differences in background characteristics between the monthly and quarterly dosing groups among EM patients. However, compared to the CM monthly dosing group, the CM quarterly dosing group exhibited significantly more photophobia as well as non-significant trends to have more osmophobia, allodynia, past prophylactic drug failures, and cases switched from other CGRP monoclonal antibodies. The effects of two different fremanezumab doses (monthly and quarterly) on MMD reduction were analyzed by two-way ANOVA using GLMM. In the EM group, there was a significant difference in time (*F* = 104.2, *p* < 0.001), but there was no difference between dosing groups (*F* = 1.08, *p* = 0.304) or interaction between time and dosing (*F* = 1.73, *p* = 0.223) (Figure 4). In the CM group, a significant difference was found in time (*F* = 82.98, *p* < 0.001), but no difference was found between dosing groups (*F* = 1.11, *p* = 0.295) or the interaction between time and dosing (*F* = 1.71, *p* = 0.116) (Figure 4). The results showed that quarterly and monthly fremanezumab doses were similarly effective in reducing MMD in the EM and CM groups.

#### Switching from other CGRP monoclonal antibodies to fremanezumab

**Figure 5:**
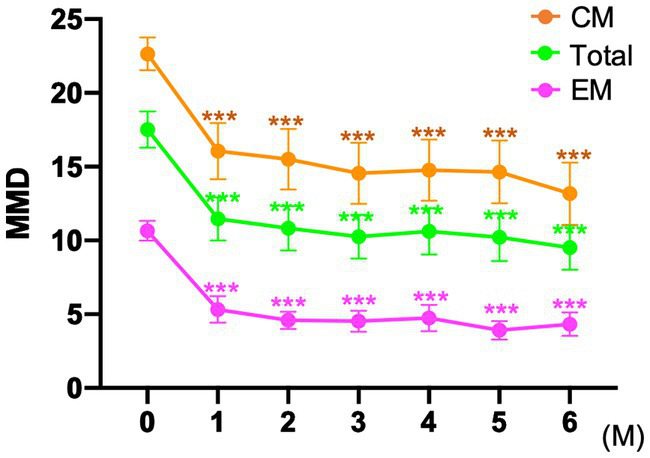
MMD changes in patients switched to fremanezumab from other CGRP monoclonal antibodies ***p < 0.001, compared to baseline using a generalized mixed-effects model with repeated measures followed by Bonferroni’s multiple comparison test. MMD, monthly migraine days; EM, episodic migraine; CM, chronic migraine.

Subanalysis was performed only for 35 patients who switched from other CGRP monoclonal antibodies to fremanezumab. Of the 35 patients, 23 (65.7%) patients were switched from galcanezumab, 4 (11.4%) from erenumab, and 8 (22.9%) from galcanezumab and erenumab. In total, in the EM and CM groups, the MMD value significantly decreased from baseline to 1, 2, 3, 4, 5, and 6 months according to one-way ANOVA using the GLMM model followed by Bonferroni’s tests (Figure 5). Patients were classified by monthly or quarterly fremanezumab dosing, and two-way ANOVA using the GLMM was performed. In the EM group, there was a significant difference in time (*F* = 22.21, *p* < 0.001), but there was no difference between dosing groups (*F* = 0.54, *p* = 0.477) or interactions of time and dosing (*F* = 2.11, *p* = 0.063) (Supplementary Figure 1). In the CM group, there was a significant difference in time (*F* = 20.89, *p* < 0.001), but there was no difference between dosing groups (*F* = 2.38, *p* = 0.141) or interactions of time and dosing (*F* = 1.11, *p* = 0.363) (Supplementary Figure 1).

#### Safety

Adverse reactions were observed in 12 (9.5%) of the total patients. The most common adverse reaction was injection site reaction (*n* = 11, 8.6%). One patient (0.8%) had constipation. Except for one patient who dropped out because of injection site reaction, all adverse reactions were mild, and none required additional treatment or procedures.

## Discussion

We conducted a retrospective, single-center, observational study including patients with EM or CM who were resistant to at least one preventive therapy and assessed the efficacy of treatment with the CGRP monoclonal antibody fremanezumab for 6 months. Our main findings are that fremanezumab significantly reduced the MMD values in total, EM and CM patients over 6 months, and the effects of monthly and quarterly fremanezumab dosing on MMD reductions were comparable.

Although the CM group exhibited more MMD reductions than the EM group, reflecting the number of migraine days at baseline, the MMD response rate was more favorable in the EM group in our study. As shown in Figure 3, response rates of ≥50% at 1, 3, and 6 months were 31.1, 52.7, and 52.2% in the CM group, while response rates of ≥50% at 1, 3, and 6 months were 65.5, 80.0, and 90.4% in the EM group, respectively. In a clinician panel–based retrospective chart review including 1,003 patients with migraine, the reduction in MMD values at 6 months was −7.7 and −10.1 days, respectively, and ≥ 50% reduction at 6 months in MMD was 75.8 and 76.3% in the EM and CM groups, respectively (8). In a recent multicenter study with a 6-month follow-up after fremanezumab treatment that included 410 high-frequency EM or CM patients, the proportions of ≥50%, ≥75, and 100% responders at 6 months were 74–78%, 40–46%, and 4–5%, respectively (10). In our study, the overall MMD reduction rate was comparable, and in the EM group, the MMD reduction rate at 6 months was superior to that in previous studies (7, 8, 10). The study reported by Driessen et al. (8) is an excellent large study encompassing a variety of complications, but at the endpoint of 6 months, the substantial number of patients in the analysis was reduced from baseline. In contrast, in our study, data from 110 (86.6%) of 127 patients were assessable at the endpoint of 6 months. McAllister et al. (9) reported that fremanezumab reduced the patient-reported average number of headache days per month by 14 days (−63%) while also providing attenuation of headache intensity and reduction in healthcare resource utilization in a clinical setting. We also observed that 65.1% of patients with CM experienced remission to EM after 6 months, and 57.1% continued to have EM remission between 4 and 6 months. In agreement with our findings, after fremanezumab treatment, remission rates of 50–75% from CM to EM have been reported from *post hoc* analysis of RCTs and real-world studies (7, 16).

Some (7, 9), but not all (8), real-world studies of fremanezumab excluded patients with a history of other CGRP monoclonal antibody treatments. In our study, 35 patients (27.6%) were switched from other CGRP monoclonal antibodies to fremanezumab. In a subanalysis including only patients who switched from other CGRP monoclonal antibodies, the MMD value significantly decreased from baseline to 1–6 months. We believe that these results are meaningful in clinical practice, where patients do not always respond well to other CGRP monoclonal antibodies. However, an open-label extension study after a 3-month RCT showed that headache days continued to decrease gradually over a period of 12 months after treatment with galcanezumab (17). This result may suggest that providing a prolonged period of CGRP antagonism, rather than switching between CGRP monoclonal antibodies, may be important in attenuating headache for some patients.

In previous studies, several predictors of the efficacy of treatment with CGRP monoclonal antibodies have been identified, such as younger age (7), normal weight, unilateral pain, good response to triptan (18), comorbid hypertension, specific allelic variants in calcitonin receptor-like receptor (19) and unilateral cranial autonomic symptoms (20). We evaluated clinical factors contributing to a ≥ 50% reduction in MMD value at 6 months. There were no significant differences in age, body mass index, sensory hypersensitivities, accompanying symptoms, or location or characteristics of pain at baseline. However, EM, lower MMD values and a higher percentage of nausea at baseline were associated with a ≥ 50% MMD reduction at 6 months. In a real-world study of galcanezumab, ≥50% responders at month 3 had more accompanying symptoms, such as nausea and vomiting, than nonresponders (21). Gastrointestinal symptoms such as nausea may be caused by elevated CGRP levels (22). Thus, nausea may be an accompanying symptom that predicts the effect of CGRP monoclonal antibodies that antagonize CGRP. The number of preventive medication classes taken previously did not affect the ≥50% reduction in MMD value at 6 months, suggesting that fremanezumab could be an option for patients who have failed several types of previous prophylactic treatments.

In Japan, switching from galcanezumab or erenumab to fremanezumab is possible in the case of inadequate efficacy or poor tolerability. The efficacy of fremanezumab should be evaluated after 3 months for monthly dosing and after 3 or 6 months for quarterly dosing, and if the treatment is effective, continuation is recommended thereafter. However, there are no clear guidelines in Japan for how long CGRP monoclonal antibody treatment should be continued, and the decision depends on the physician’s judgment. Recently, European guidelines have included a statement that the efficacy of CGRP monoclonal antibodies should be determined after at least 3 months of use (23), but real-world data on how exactly to determine the efficacy of CGRP monoclonal antibodies are not yet available. Thus, we evaluated whether the ≥50% response at month 6 could be predicted by the ≥50% efficacy in months 1, 2, and 3 alone, the average ≥ 50% response rate in months 1–3 or at least one ≥50% response rate in any of months 1–3. We found that at least one ≥50% response rate in any of months 1–3 was a better predictor of ≥50% response at month 6, with a sensitivity of 90.5% and specificity of 80.6% compared with the other criteria. This finding that at least one ≥50% response rate in any of months 1–3 could be a better predictor of favorable outcome at 6 months is very important to motivate patients to continue CGRP monoclonal antibodies in a clinical setting where the MMD value is susceptible to climate change, stress, and other factors depending on each patient. In line with our results, a long-term open-label study showed that a ≥ 50% reduction in MMDs at month 3 after initiation of erenumab treatment could predict 1-year outcome, but combinations of ≥50% response rates at 1–3 months were not evaluated (24).

In our study, the effects of two different types of dosing on MMD reduction over 6 months were analyzed by two-way ANOVA using GLMM. The results indicated that fremanezumab quarterly and monthly dosing were similarly effective in the EM and CM groups. The CM quarterly group tended to have more osmophobia, allodynia, and previous prophylaxis failure compared with the CM monthly group, suggesting that patients with severe symptoms who had difficulty visiting the hospital every month preferred quarterly dosing, although baseline MMD between the CM quarterly and monthly dosing groups did not differ.

In the present study, adverse reactions were similar to those seen in other real-world studies of CGRP monoclonal antibodies (7, 25), with injection site reactions being more common and less severe. Only one patient discontinued fremanezumab due to injection site reaction; none of the remaining patients discontinued due to side effects.

The strengths of this study are as follows: (1) patients with various comorbidities, including MOH and those switching from other CGRP monoclonal antibodies, were included; and (2) the MMD vale was assessed based on individual patient headache diaries. The limitations of the study are that the observation period was limited to 6 months, and we did not evaluate changes in accompanying symptoms, hypersensitivity symptoms, blood pressure or migraine-related disability before and after treatment with fremanezumab. In some cases, the side effects of some prophylaxis drugs led to immediate discontinuation, and the effect of each previously used preventive drug was not consistently monitored over a 3-month period. In our study, the CM group had an average MMD of 21.7 ± 4.8 and fewer comorbid MOH (24.7%), which may have influenced the study results. Because of the small sample size of patients who switched from other CGRP monoclonal antibodies to fremanezumab (*n* = 35), we did not assess the difference in clinical efficacy between the switch from erenumab to fremanezumab and the switch from galcanezumab to fremanezumab. Further research is needed to elucidate the clinical factors that support the recommendation of fremanezumab for patients who have failed other CGRP monoclonal antibodies.

In conclusion, the results of our real-world study confirmed that prophylactic treatment with monthly or quarterly fremanezumab showed both favorable efficacy and tolerability in patients with EM or CM over 6 months.

## Data availability statement

The raw data supporting the conclusions of this article will be made available by the authors, without undue reservation.

## Ethics statement

The studies involving human participants were reviewed and approved by the Institutional Review Board of the Dokkyo Medical University Hospital. Written informed consent for participation was not required for this study in accordance with the national legislation and the institutional requirements.

## Author contributions

SS and KS drafted the manuscript. KS and YH performed the statistical analysis. TS and KH performed a critical review for important intellectual content. All authors contributed to the acquisition and interpretation of data for this study and approved the final manuscript.

## Conflict of interest

SS, TS, and KH received lecture fees from Eli Lilly Japan, Daiichi Sankyo, Amgen, and Otsuka Pharmaceutical Co., Ltd., during the conduct of the study. KS received lecture fees from Eli Lilly Japan, Daiichi Sankyo, and Otsuka Pharmaceutical Co., Ltd., during the conduct of the study.

The remaining author declares that the research was conducted in the absence of any commercial or financial relationships that could be construed as a potential conflict of interest.

## Publisher’s note

All claims expressed in this article are solely those of the authors and do not necessarily represent those of their affiliated organizations, or those of the publisher, the editors and the reviewers. Any product that may be evaluated in this article, or claim that may be made by its manufacturer, is not guaranteed or endorsed by the publisher.
