# Supplementary material for: Real-world experience with monthly and quarterly dosing of fremanezumab for the treatment of patients with migraine in Japan
Source: Front Neurol. 2023 Jul 6;14:1220285. doi: 10.3389/fneur.2023.1220285 (PMC10359132; doi:10.3389/fneur.2023.1220285)
Supplement: Supplementary file 1 [file Data_Sheet_1.PDF]

Supplementary Table 1: Comorbidities of patients with migraine

	Total	EM	CM	*p* value
Comorbidities, n (%)	69 (54.3)	20 (37.0)	49 (67.1)	<0.001
Neurological disorder, n (%)	3 (2.4)	2 (3.7)	1 (1.4)	0.392
Cardiovascular disorder, n (%)	28 (22)	7 (13.0)	21 (28.8)	0.034
Endocrine disorder, n (%)	4 (3.1)	0 (0.0)	4 (5.5)	0.080
Respiratory disease, n (%)	7 (5.5)	1 (1.9)	6 (8.2)	0.120
Connective tissue disease, n (%)	3 (2.4)	2 (3.7)	1 (1.4)	0.392
Sleep disorder, n (%)	9 (7.1)	4 (7.4)	5 (6.8)	0.904
Digestive disorder, n (%)	5 (3.9)	1 (1.9)	4 (5.5)	0.299
Psychiatric disorder, n (%)	12 (9.4)	3 (5.6)	9 (12.3)	0.197
Hematological disorder, n (%)	3 (2.4)	0 (0.0)	3 (4.1)	0.132
Gynecological disorder, n (%)	4 (3.1)	2 (3.7)	2 (2.7)	0.758
Others, n (%)	8 (6.3)	3 (5.6)	5 (6.8)	0.767

EM, episodic migraine; CM, chronic migraine

Supplementary Table 2: Baseline characteristics of patients with migraine receiving fremanezumab monthly and quarterly

	EM			CM		
Fremanezumab dosing	Monthly	Quarterly	*p* value	Monthly	Quarterly	*p* value
n (M/F)	30 (5/25)	24 (9/15)	0.083	45 (10/35)	28 (7/21)	0.785
Age, years	42.5±12.7	46.1±12.7	0.303	45.7±13.4	46.6±11.5	0.774
Body mass index (kg/m^2)	21.9±4.1	22.5±3.6	0.613	22.6±3.9	23.1±3.4	0.587
Migraine with aura, n (%)	6 (20.0)	9 (37.5)	0.154	6 (13.3)	5 (17.9)	0.599
Medication overuse headache, n (%)	0 (0.0)	0 (0.0)	-	10 (22.2)	8 (28.6)	0.541
Disease duration, years	21.9±10.6	25.0±11.0	0.294	26.8±12.2	29.3±10.8	0.396
Pain location, n (%)						
Unilateral	16 (53.3)	17 (70.8)	0.190	28 (62.2)	23 (82.1)	0.071
Bilateral	22 (73.3)	18 (75.0)	0.890	34 (75.6)	18 (64.3)	0.301
Pain characteristics, n (%)						
Pulsating	28 (93.3)	24 (100)	0.197	35 (77.8)	27 (96.4)	0.030
Pressing	19 (63.3)	15 (62.5)	0.950	28 (62.2)	20 (71.4)	0.420
Others	1 (3.3)	0 (0.0)	0.367	3 (6.7)	0 (0.0)	0.163
Sensory hypersensitivity, n (%)						
Photophobia	26 (86.7)	20 (83.3)	0.732	31 (68.9)	26 (92.9)	0.016
Phonophobia	23 (76.7)	17 (70.8)	0.627	32 (71.1)	22 (78.6)	0.480
Osmophobia	19 (63.3)	12 (50.0)	0.325	15 (33.3)	15 (53.6)	0.087
Nausea	29 (96.7)	23 (95.8)	0.872	38 (84.4)	25 (89.3)	0.559
Allodynia	6 (20.0)	6 (25.0)	0.661	5 (11.1)	8 (28.6)	0.058
Number of preventive medication classes taken previously, n (%)			0.689			0.052
1	9 (30.0)	11 (45.8)		9 (20.0)	8 (28.6)	
2	14 (46.7)	9 (37.5)		15 (33.3)	6 (21.4)	

3	5 (16.7)	3 (12.5)		13 (28.9)	6 (21.4)	
4	2 (6.7)	1 (4.2)		6 (13.3)	1 (3.6)	
≥5	0 (0.0)	0 (0.0)		2 (4.4)	7 (25.0)	
Baseline MMD, n (%)	10.8±2.4	10.5±2.7	0.657	22.1±4.6	21.1±5.0	0.395
Comorbidities, n (%)	9 (30.0)	11 (45.8)	0.268	29 (64.4)	20 (71.4)	0.537
Switching from other CGRP mAb to fremanezumab, n (%)	8 (26.7)	7 (29.2)	0.839	9 (20.0)	11 (40.7)	0.057

EM, episodic migraine; CM, chronic migraine; CGRP mAb, calcitonin gene-related peptide antibodies monoclonal antibodies

Supplementary Figure legends

Supplementary Figure 1: Mean changes from baseline in monthly migraine days in patients with EM or CM switched to fremanezumab from other CGRP monoclonal antibody patients receiving monthly or quarterly dosing of fremanezumab

EM group: $F=22.21$, $p<0.001$ in time; $F=0.54$, $p=0.477$ in dosing; and $F=2.11$, $p=0.063$ in interaction between time and dosing.
CM group: $F=20.89$, $p<0.001$ in time; $F=2.38$, $p=0.141$ in dosing; and $F=1.11$, $p=0.363$ in interaction between time and dosing.
A generalized mixed-effects model with repeated measures followed by a global test was used.
MMD, monthly migraine days; EM, episodic migraine; CM, chronic migraine

Supplementary Figure 1

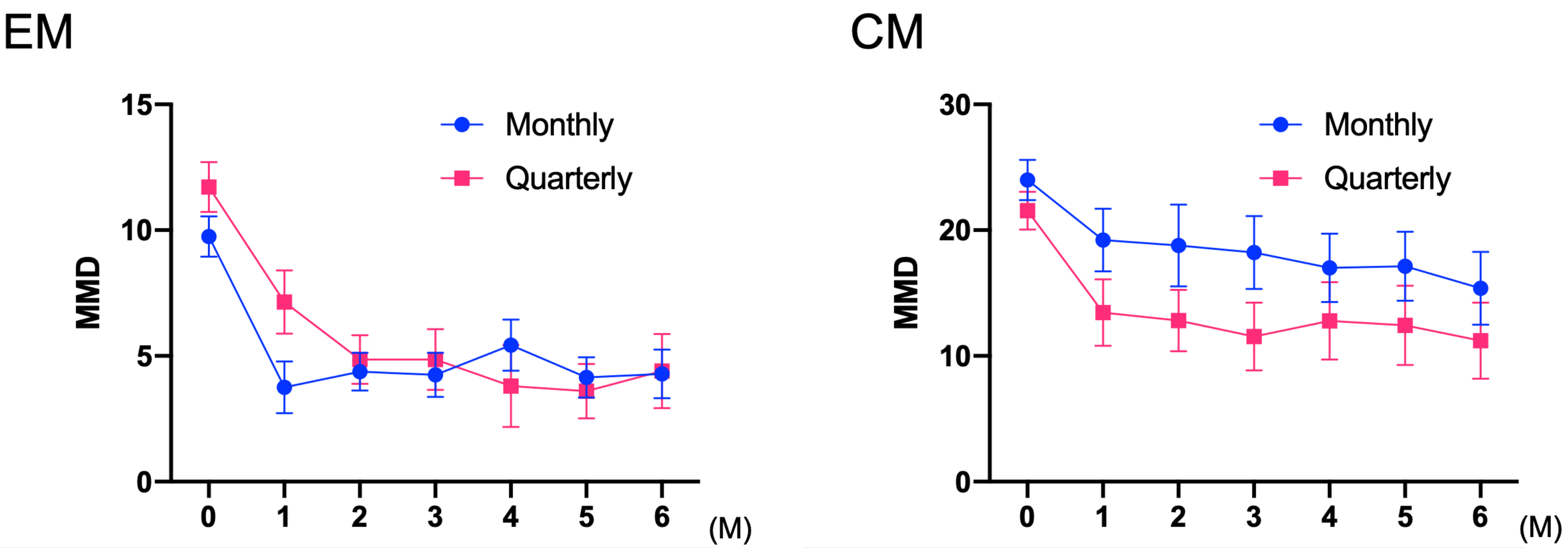

Monthly, n	8	8	8	8	7	7	7
Quarterly, n	7	7	7	7	5	5	5

Monthly, n	9	9	9	9	8	8	8
Quarterly, n	11	11	11	11	9	9	9
